# Implementing policies and predictive stochastic models to restrict borderline personality disorder’s access to restricted medications: comorbidity with factitious disorder, functional neurological disorder and medically unexplained symptoms

**DOI:** 10.1192/j.eurpsy.2024.1004

**Published:** 2024-08-27

**Authors:** C. G. Lazzari

**Affiliations:** Community Mental Health, UK NHS, BRIGHTON, United Kingdom

## Abstract

**Introduction:**

We are facing increased access to hospital beds and increased use of restricted medications by people with borderline personality disorder (BPD). Our former research shows BPD comorbidity with factitious conditions, functional neurological disorder and medically unexplained symptoms. We also registered that persons with BPD might craft or exaggerate symptoms to access restricted medications. In the worst cases, they might share these medications (benzodiazepines, hypnotics, and anxiolytics) with street values for profit or other recreational purposes.

**Objectives:**

To generate forecasting models and preventive policies to deal with BPD factitious disorders and improve the effectiveness of the UK National Healthcare Service (NHS) in reducing unnecessary admissions to general and psychiatric hospitals. More selective policies will capture and discourage BPD’s feigning and exaggerating symptoms for accessing restricted medications.

**Methods:**

The underlying analysis framework is stochastic forecasting. We used current knowledge and data to complete systematic future predictions extracted from recent trends. A logical-mathematical model generated the required expressions. We identify four major model components to be introduced in the model: BPD (A), factious disorders (B), prescribing restricted medications (C), antisocial behaviours (D), and access to hospital beds (E).

**Results:**

The Boolean expression becomes [A then B then C then D then E], or [A ⇒ (B ⇒ (C ⇒ (D ⇒ E))] with a truth density of 96.875% (Figure 1).

**Conclusions:**

BPD should alert healthcare of the risks of symptom exaggeration and factitious mental diseases. These conditions are used to access often restricted medications, such as benzodiazepines, sleep tablets, and anxiolytics, for personal and communal use. Street sharing of these last increases local criminality. In worst cases, a hospital bed is granted without preventive triage. The risk is the indoor access to these medications. We advocate policies for the discontinuation of community prescription of these drugs.

**Disclosure of Interest:**

None Declared

